# Effects of SDF-1/CXCR7 on the Migration, Invasion and Epithelial-Mesenchymal Transition of Gastric Cancer Cells

**DOI:** 10.3389/fgene.2021.760048

**Published:** 2021-11-09

**Authors:** Ameng Shi, Ting Wang, Miao Jia, Lei Dong, Haitao Shi

**Affiliations:** ^1^ Department of Ultrasound, The Second Affiliated Hospital of Xi’an Jiaotong University, Xi’an, China; ^2^ Department of Gastroenterology, The Second Affiliated Hospital of Xi’an Jiaotong University, Xi’an, China

**Keywords:** chemokine (CXC motif) receptor 7 (CXCR7), migration, invasion, epithelial-mesenchymal transition (EMT), gastric cancer

## Abstract

We found that SDF-1/CXCR7 axis played an important role in the growth and proliferation of gastric cancer in the previous studies. The objectives of this study were to explore the effects of SDF-1/CXCR7 on the metastatic ability of gastric cancer cells and the possible mechanisms. CXCR7 expression in SGC-7901 gastric cancer cells was stably knocked down via lentiviral vectors. The cell migration and invasion abilities were detected by transwell migration and invasion assays. The expressions of matrix metalloproteinase 2 (MMP-2), MMP-9, vascular endothelial growth factor (VEGF), epithelial-mesenchymal transition (EMT) markers and Akt phosphorylation were detected with real-time PCR and/or western blot. We found that SDF-1 markedly enhanced the migration and invasion abilities of SGC-7901 gastric cancer cells; CXCR7 knockdown inhibited these effects. SDF-1/CXCR7 increased the expressions of MMP-2, MMP-9 and VEGF. SDF-1/CXCR7 also downregulated E-cadherin expression but upregulated N-cadherin, vimentin and Snail expressions, suggesting that SDF-1/CXCR7 could promote the development of EMT in gastric cancer cells. Furthermore, SDF-1/CXCR7 could promote Akt phosphorylation. Our results indicated that SDF-1/CXCR7 enhanced the migration, invasion and EMT of gastric cancer cells and thus CXCR7 supression may be a strategy for inhibiting gastric cancer metastasis.

## Introduction

According to Global Cancer Statistics 2018, gastric cancer is the fifth most common malignant tumor worldwide (Bray et al., 2018). Surgery, chemotherapy, radiotherapy, molecular targeted therapy and immunotherapy are still the major treatment methods currently. The prognosis of patients with progressed gastric cancer is relatively poor. Even with surgery, the survival rate is still lower than 30% (Irino et al., 2021). Therefore, it’s still a challenge to clarify the molecular mechanisms of gastric cancer and search for new therapeutic targets.

CXCL12, also known as stromal derived factor-1 (SDF-1), has two receptors, CXCR4 and CXCR7. Many studies have found that SDF-1/CXCR4 axis regulates gastric cancer proliferation, migration, invasion, metastasis and angiogenesis (Xue et al., 2017). CXCR7 is discovered in recent years and lacks the classical DRYLAIV motif and cannot mediate Gαi protein activation (Graham et al., 2012). CXCR7 has significantly higher affinity than CXCR4 for SDF-1. CXCR7 and CXCR4 can each form homodimers and heterodimers. When CXCR7 and CXCR4 were co-expressed, both of the two receptors could be activated in the presence of SDF-1. Therefore, the role of SDF/CXCR7 in pathophysiology has been getting more attention gradually. CXCR7 expression is upregulated in malignant tumors; CXCR7 affects tumor growth and metastasis and is associated with poor prognosis. We have long focused on the role of CXCR7 in gastric cancer. Our previous study found that the increased expression of CXCR7 in gastric cancer tissues was correlated with tumor size and lymph node metastasis. In addition, SDF-1/CXCR7 accelerated the proliferation of SGC-7901 gastric cancer cells and the extracellular signal-regulated kinase1/2 (ERK1/2) and p38 signaling pathways might be involved these effects (Shi et al., 2017). However, its effects on the metastasis of gastric cancer cells and relevant mechanisms especially the Epithelial-mesenchymal transition (EMT) are not clear. Existing researches revealed that CXCR7 can increase the migration and invasion abilities of some malignant tumors *in vitro*, including liver cancer (Zheng et al., 2010; Lin et al., 2014; Neve Polimeno et al., 2015), pancreatic cancer (Guo et al., 2016), colon cancer (Xu et al., 2011), kidney cancer (Ierano et al., 2014), breast cancer (Wani et al., 2014), ovarian cancer (Yu et al., 2014), prostate cancer (Wang et al., 2008; Yang et al., 2018), bladder cancer (Hao et al., 2012), and glioma (Liu et al., 2013). However, the related mechanism is not very distinct. EMT and angiogenesis are important factors in tumor metastasis and targeting them is a potential therapeutic strategy. Therefore, the current research is mainly to explore the effects of SDF-1/CXCR7 on the migration, invasion, angiogenesis and EMT of gastric cancer cells *in vitro*.

## Materials and Methods

### Cells Culture

SGC-7901, a moderately differentiated human gastric adenocarcinoma cell line, was purchased from the Cell Bank of Chinese Academy of Sciences (Shanghai, China) and used in our previous study (Shi et al., 2017). The cells were incubated in RPMI 1640 medium (HyClone, Logan, UT, United States) supplemented with 10% fetal bovine serum (Sijiqing, Hangzhou, China) at 37°C in 5% CO_2_ incubator.

### CXCR7 Knockdown

Three shRNA-expressing lentiviral vectors (LV) including LV-negative control (LV-NC), LV-CXCR7-1 and LV-CXCR7-2 were from Cyagen Biosciences. CXCR7-siRNA-1 was 5′-CGC​ACT​GCT​ACA​TCT​TGA​A-3′, CXCR7-siRNA-2 was 5′-GCC​GTT​CCC​TTC​TCC​ATT​ATC-3′. In the presence of polybrene (5 μg/ml), the SGC-7901 cells infected with these three kinds of LV were selected by puromycin (1.5 μg/ml) (Sigma-Aldrich, Saint Louis, MO, United States). Reverse transcription quantitative real-time PCR (RT-qPCR) and western blot were used to detected the mRNA and protein expressions.

### Reverse Transcription Quantitative Real-Time PCR (RT-qPCR)

RNA extraction kit (Fastagen, Shanghai, China) was used to extract total RNA according to the manufacturer’s instruction. The RNA purity and concentration were measured with Thermo Scientific NanoDrop spectrophotometer. cDNA was synthesized according to the instructions of PrimeScriptTM RT Master Kit (TaKaRa, Otsu, Japan). The required primer sequences are listed in Table 1. SYBR Premix Ex TaqTM II kit (TaKaRa, Otsu, Japan) was used to perform RT-qPCR according to the manufacturer’s protocol. The applied PCR conditions were: preliminary denaturation at 95°C for 30 s, followed by 40 cycles at 95°C for 5 s and 60°C for 30 s. The 2^−△△CT^ (CT: threshold cycle) method was used to calculate the RNA expression levels.

**TABLE 1:** Primer sequences for real-time PCR.

Target genes	Sequences	
β-actin	Forward	5′-ATC​GTG​CGT​GAC​ATT​AAG​GAG​AAG-3′
	Reverse	5′-AGG​AAG​GAA​GGC​TGG​AAG​AGT​G-3′
CXCR7	Forward	5′-CCT​GAC​ACC​TAC​TAC​CTG​AAG​AC-3′
	Reverse	5′-CAC​TGG​ACG​CCG​AGA​TGG-3′
E-cadherin	Forward	5′-ACC​AAC​GAT​AAT​CCT​CCG​AT-3′
	Reverse	5′-TCA​GTG​TGG​TGA​TTA​CGA​CG-3′
N-cadherin	Forward	5′-AAT​CCT​CCA​GAG​TTT​ACT​GC-3′
	Reverse	5′-TCC​TTA​TCG​GTC​ACA​GTT​AG-3′
Vimentin	Forward	5′-GAG​AGG​AAG​CCG​AAA​ACA​C-3′
	Reverse	5′-TGC​GTT​CAA​GGT​CAA​GAC​G-3′
Snail	Forward	5′-TTA​CCT​TCC​AGC​AGC​CCT​AC-3′
	Reverse	5′-AGA​GTC​CCA​GAT​GAG​CAT​TG-3′
MMP-2	Forward	5′-GAC​CAC​AGC​CAA​CTA​CGA​TG-3′
	Reverse	5′-ACG​GAA​GTT​CTT​GGT​GTA​GG-3′
MMP-9	Forward	5′-ACC​TCG​AAC​TTT​GAC​AGC​GAC​A-3′
	Reverse	5′-GAT​GCC​ATT​CAC​GTC​GTC​CTT​A-3′
VEGF	Forward	5′-GGG​CAG​AAT​CAT​CAC​GAA​GT-3′
	Reverse	5′-GAA​GAT​GTC​CAC​CAG​GGT​CT-3′

### Western Blot

RIPA lysis buffer (Beyotime, China) was used to extract total protein according to the manufacturer’s instruction. The total protein sample was separated by SDS-PAGE, transferred to a PVDF membrane (Millipore, Billerica, MA, United States), blocked in non-fat milk, incubated with primary antibodies at suitable dilution concentration followed by secondary antibody. The protein bands were detected using an ECL plus chemiluminescence detection kit (Millipore, Billerica, MA, United States). Gel-pro Analyzer 4.0 software (Media Cybernetics, CA, United States) was used to analyze the integral optical density (IOD). Polyclonal antibodies against CXCR7, MMP-2, MMP-9, VEGF, E-cadherin, N-cadherin, vimentin, snail, β-actin and secondary antibody were purchased from Santa Cruz, Dallas, TX, United States. Monoclonal antibodies against p-Akt and t-Akt were purchased from Cell Signaling Technology, Danvers, MA, United States.

### Cell Migration Assay

Serum starved LV-NC and LV-CXCR7-1 cells were resuspended at a cell density of 5 × 10^5^ cells/ml in medium containing 1% FBS. 200 μl of the cell suspension was added to the upper chamber of a 24-well transwell, and 600 μl of medium containing 10% FBS with or without SDF-1 (100 ng/ml) was added into the lower chamber. There were four experimental groups: LV-NC, LV-NC + SDF-1, LV-CXCR7-1 and LV-CXCR7-1+SDF-1. After 48 h of incubation, the cells were washed with PBS, fixed with methanol for 10 min and washed with PBS again. The chamber was stained with crystal violet for 20 min and then washed with PBS. The cells on the upper transwell chamber surface that failed to perforate the chamber were gently wiped off with a cotton swab. The perforating cells on the lower chamber surface were observed and pictured under an inverted microscope (Nikon, Japan). Five fields of view were randomly selected to count the number of perforating cells; the average count was calculated.

### Cell Invasion Assay

50 μl of diluted Matrigel (BD Biosciences, Bedford, MA, Unites States) was added to the upper chamber of the transwell. The chamber was cultured overnight at 37°C in a 5% CO_2_ incubator to reconstitute the basement membrane. This assay simulates the invasion process of tumor cells, which is different from the migration assay. In cell invasion assay, gastric caner cells need to break down matrigel to penetrate the basement membrane. On the day after basement membrane reconstruction, serum-starved LV-NC and LV-CXCR7-1 cells were resuspended at cell density of 5 × 10^5^ cells/ml in medium containing 1% FBS; 200 μl of the cell suspension was added to the upper chamber of a 24-well transwell coated with Matrigel, and 600 μl of medium containing 10% FBS with or without SDF-1 (100 ng/ml) was added to the lower chamber. The culture plates were transferred to an incubator for 48 h. Thereafter, the procedures were the same as those described for the cell migration assay. The number of perforating cells among the four groups was compared.

### Statistical Analysis

SPSS version 13.0 (SPSS Inc., Chicago, IL, United States) was used for statistical analysis. The data were shown as the mean ± standard deviation (SD). One-way ANOVA and LSD-t test were employed to compare between-group comparisons. It was considered to be significant different when *p* < 0.05.

## Results

### CXCR7 Knockdown in SGC-7901 Gastric Cancer Cells

**FIGURE 1:**
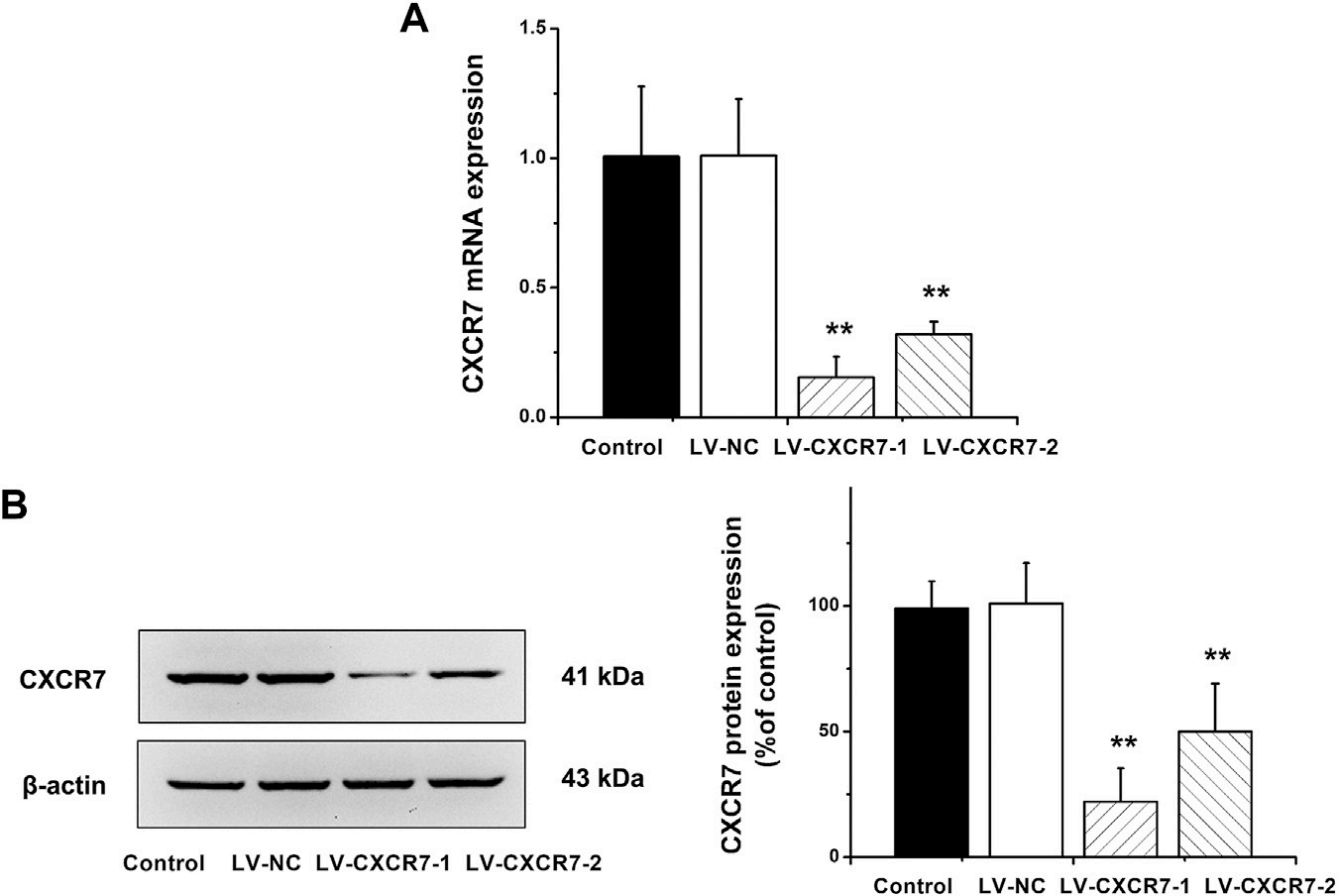
The knockdown of CXCR7 in gastric cancer cells. **(A)** CXCR7 mRNA expression was detected by RT-qPCR. **(B)** CXCR7 protein expression was detected by western blot. Data are shown as mean ± SD. Origin 7.0 software (OriginLab Corporation, United States) was used for creation of histogram. **p < 0.01 vs. LV-NC group.

CXCR7 was knockdown using the shRNA-expressing LV. LV-NC did not change the expression of CXCR7. The mRNA and protein expression of CXCR7 were significantly lower in LV-CXCR7-1 transfected cells compared with Control or LV-NC or LV-CXCR7-2 transfected cells (all *p* < 0.01). (Figure 1). Therefore, LV-CXCR7-l cells were used in the following experiments.

### SDF-1/CXCR7 Promoted the Migration and Invasion Abilities of Gastric Cancer Cells

**FIGURE 2:**
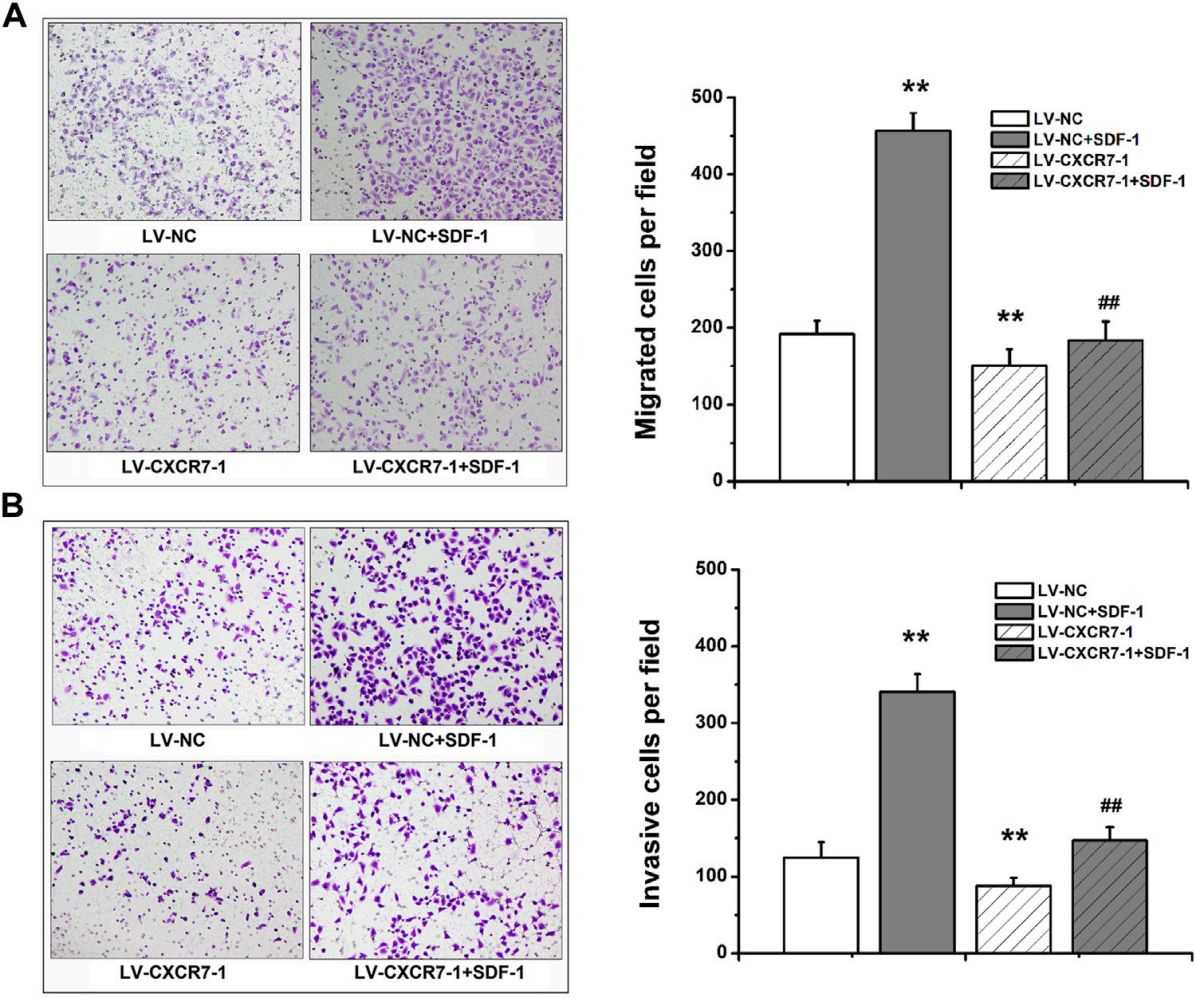
The effects of SDF-1/CXCR7 on the migration and invasion abilities of gastric cancer cells. **(A)** Transwell migration assay was used to evaluate the migration abilities. The perforating cells were stained with crystal violet and pictured under an inverted microscope (200×) (Nikon, Japan). **(B)** Transwell invasion assay was used to evaluate the invasion abilities. The perforating cells were stained with crystal violet and pictured under an inverted microscope (200×) (Nikon, Japan). Data are shown as mean ± SD. Origin 7.0 software (OriginLab Corporation, United States) was used for creation of histogram. **p < 0.01 vs. LV-NC group; ^##^ *p* < 0.01 vs. LV-NC + SDF-1 group.

Transwell migration and invasion assay were used to determine the migration and invasion abilities. The number of perforating cells among the four groups was compared. The results showed that compared with that in the LV-NC group, the number of perforating cells in the LV-NC + SDF-1 group was markedly higher, and the number in the LV-CXCR7-1 group was lower. Meanwhile, the number of perforating cells in the LV-CXCR7-1 + SDF-1 group was significantly lower compared with that in the LV-NC + SDF-1 group (all *p* < 0.01). In addition, transwell invasion assay showed that SDF-1 markedly enhanced the invasion abilities of SGC-7901 gastric cancer cells and CXCR7 knockdown inhibited these effects (Figure 2).

### SDF-1/CXCR7 Promoted the EMT of Gastric Cancer Cells

**FIGURE 3:**
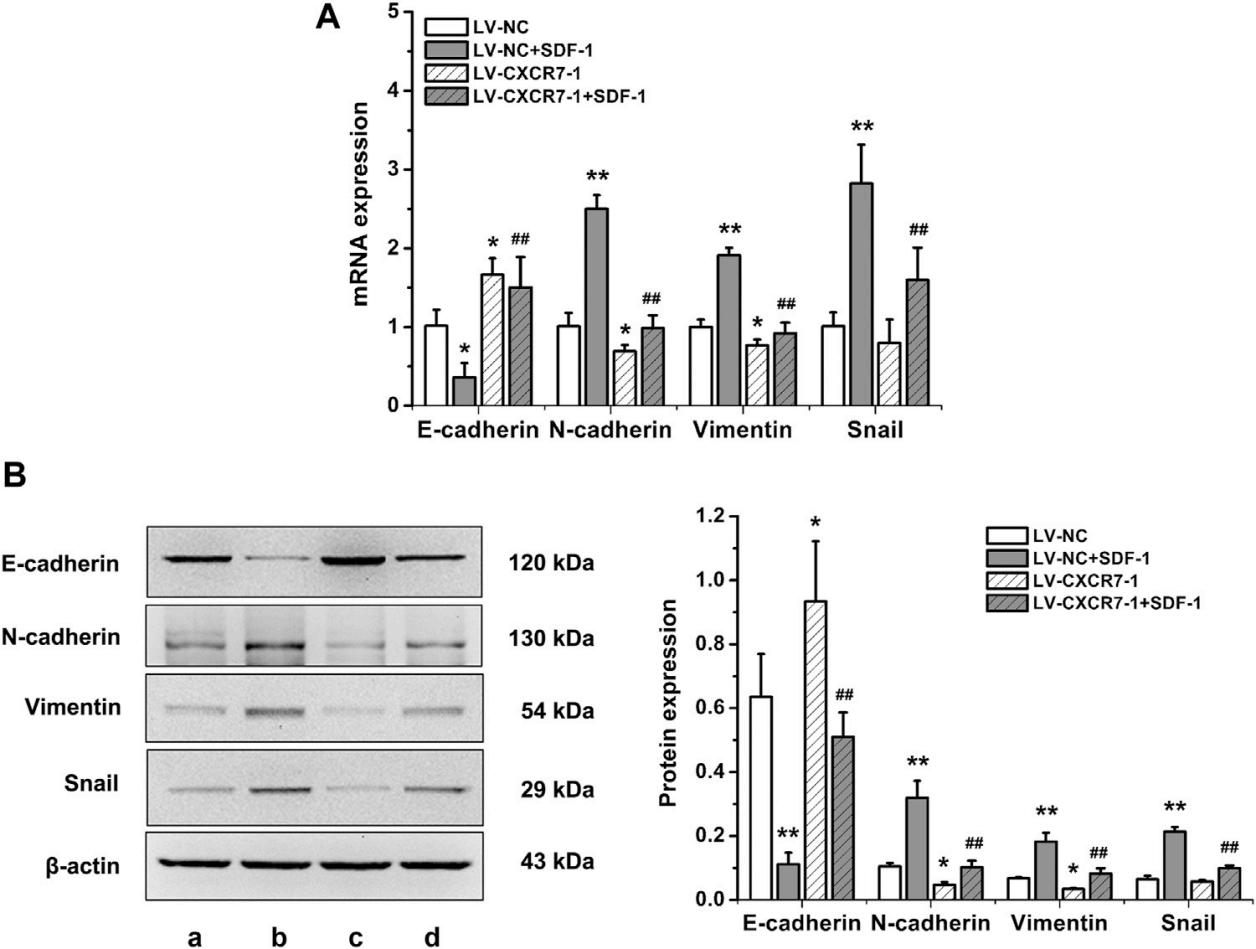
The effects of SDF-1/CXCR7 on EMT in gastric cancer cells. **(A)** The mRNA expressions of E-cadherin, N-cadherin, Vimentin and Snail were detected by RT-qPCR. **(B)** The protein expressions of E-cadherin, N-cadherin, Vimentin and Snail were detected by western blot. a: LV-NC group; b: LV-NC + SDF-1 group; c: LV-CXCR7-1 group; d: LV-CXCR7-1+SDF-1 group. Data are shown as mean ± SD. Origin 7.0 software (OriginLab Corporation, United States) was used for creation of histogram. *p < 0.05, **p < 0.01 vs. LV-NC group; ^##^ *p* < 0.01 vs. LV-NC + SDF-1 group.

To explore the effects of SDF-1/CXCR7 on the EMT of gastric cancer cells, RT-qPCR and western blot were performed to detect the mRNA and protein expression of EMT markers including E-cadherin, N-cadherin, vimentin and Snail. The results were shown in Figure 3. SDF-1 markedly decreased E-cadherin mRNA and protein expressions in LV-NC cells (*p* < 0.05 and *p* < 0.01), but increased the expressions of N-cadherin, vimentin and Snail (all *p* < 0.01). CXCR7 knockdown increased E-cadherin expressions (*p* < 0.05) but decreased the expressions of N*-*cadherin and vimentin (all *p* < 0.05); When SDF-1 was present, the change was more remarkable. CXCR7 knockdown also significantly reduced Snail levels (*p* < 0.01) with SDF-1 stimulation. These results indicated that SDF-1 could promote the EMT process in gastric cancer cells through CXCR7.

### SDF-1/CXCR7 Increased the Expressions of Matrix Metalloproteinase 2 (MMP-2), MMP-9 and VEGF in Gastric Cancer Cells

**FIGURE 4:**
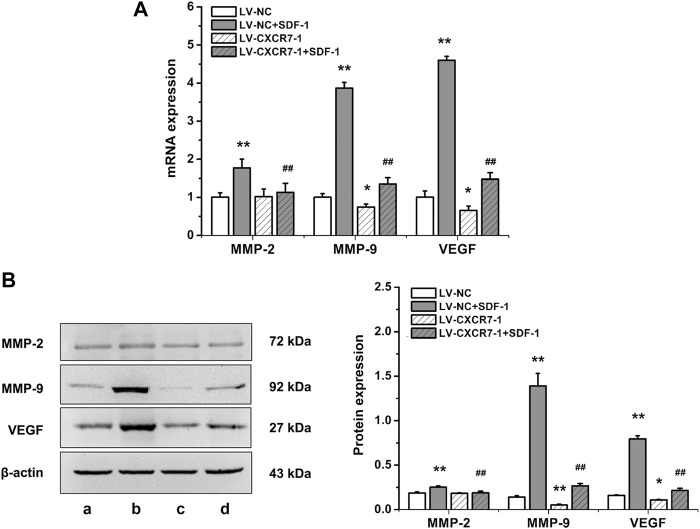
The influences of SDF-1/CXCR7 on the levels of MMP-2, MMP-9 and VEGF in gastric cancer cells. **(A)** The mRNA expressions of MMP-2, MMP-9 and VEGF were evaluated ed by RT-qPCR. **(B)** The protein expressions of MMP-2, MMP-9 and VEGF were evaluated by western blot. a: LV-NC group; b: LV-NC + SDF-1 group; c: LV-CXCR7-1 group. d: LV-CXCR7-1+SDF-1 group. Data are shown as mean ± SD. Origin 7.0 software (OriginLab Corporation, United States) was used for creation of histogram. *p < 0.05, **p < 0.01 vs. LV-NC group; ^##^ *p* < 0.01 vs. LV-NC + SDF-1 group.

To explore the mechanism of SDF-1/CXCR7 inhibiting migration and invasion, we also detected the mRNA and protein expressions of MMP-2, MMP-9 and VEGF. As shown in Figure 4, compared with that in the LV-NC group, the expressions of MMP-2, MMP-9 and VEGF in the LV-NC + SDF-1 group were significantly higher (all *p* < 0.01); MMP-2 expression in the LV-CXCR7-1 group was similar, but the expressions of MMP-9 and VEGF were lower (*p* < 0.05 and *p* < 0.01). Compared with that in the LV-NC + SDF-1 group, the expressions of MMP-2, MMP-9 and VEGF in the LV-CXCR7-1 + SDF-1 group were significantly lower (all *p* < 0.01). These results indicated that SDF-1 could increase the expressions of MMP-2, MMP-9 and VEGF in gastric cancer cells through CXCR7.

### SDF-1/CXCR7 Activated the Akt Pathway in Gastric Cancer Cells

**FIGURE 5:**
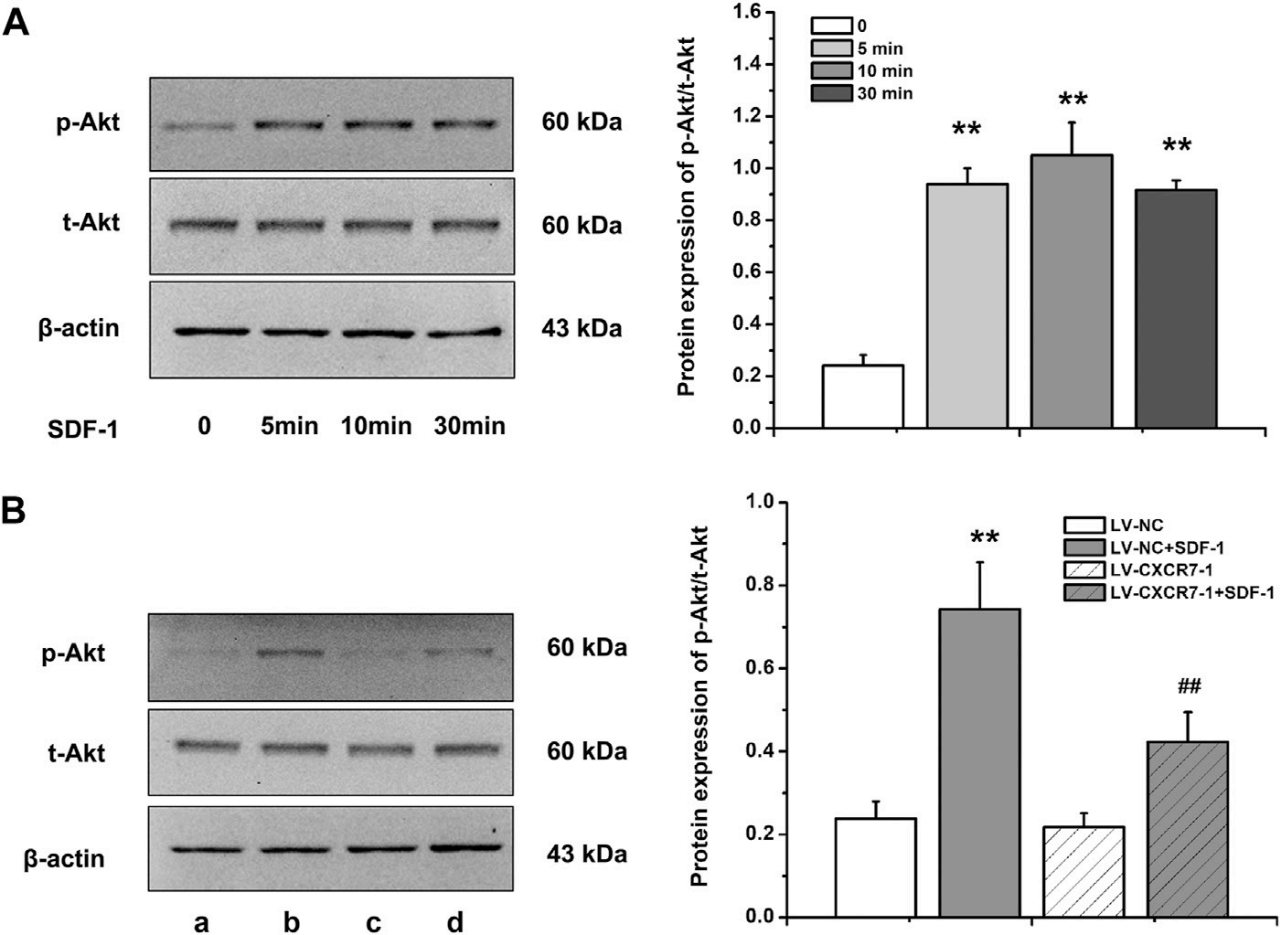
The activation of Akt pathway by SDF-1/CXCR7 in gastric cancer cells. **(A)** The protein levels of p-Akt and t-Akt after SDF-1 stimulation were detected by western blot. Data are shown as mean ± SD, **p < 0.01 vs. 0 group; **(B)** The levels of p-Akt and t-Akt after CXCR7 knockdown were detected by western blot. a: LV-NC group; b: LV-NC + SDF-1 group; c: LV-CXCR7-1 group. d: LV-CXCR7-1+SDF-1 group. Data are shown as mean ± SD. Origin 7.0 software (OriginLab Corporation, United States) was used for creation of histogram. **p < 0.01 vs. LV-NC group; ^##^ *p* < 0.01 vs. LV-NC + SDF-1 group.

Akt is a key protein in the PI3K/Akt signaling pathway and the activation of this signaling pathway is closely related to cell metastasis, angiogenesis and EMT, so we detected the expressions of p-Akt and t-Akt in gastric cancer cells. The level of p-Akt increased significantly at 5 min after SDF-1 stimulation (*p* < 0.01), and the difference of p-Akt levels was not distinct among 5, 10, and 30 min. In addition, SDF-1 stimulation did not affect t-Akt level. Therefore, gastric cancer cells were treated with SDF-1 for 10 min in the following experiments. CXCR7 knockdown significantly reduced p-Akt level (*p* < 0.01) with SDF-1 stimulation, but did not affect t-Akt level (Figure 5). These results suggest that SDF-1/CXCR7 activated the Akt signaling pathway in gastric cancer cells.

## Discussion

Gastric cancer metastasis greatly increases the difficulty of treatment and becomes the main reason for gastric cancer deaths. This process involves multiple steps, including the mesenchymal transformation of tumor cells, the separation and shedding of tumor cells, enhanced adhesion with the extracellular matrix, the degradation of extracellular matrix, migration and invasion, enhanced angiogenesis and tumor cell proliferation activity, resulting in the production of new metastatic lesions. It has been shown that CXCR7 is involved in some malignant tumors metastasis. For example, CXCR7 expression in osteosarcoma tissue was associated with tumor distant metastasis (Zhang et al., 2014). High CXCR7 expression in papillary thyroid carcinoma correlated with lymph node metastasis and distant metastasis (Dang et al., 2013). In animal experiments, it was found that CXCR7 increased lung metastasis of colon cancer and liver cancer (Guillemot et al., 2012; Lin et al., 2014) and increased liver metastasis of pancreatic cancer (Guo et al., 2016). Some studies *in vitro* have also found that SDF-1/CXCR7 enhanced the migration and invasion abilities of cancer cells, including liver cancer (Neve Polimeno et al., 2015), pancreatic cancer (Guo et al., 2016), kidney cancer (Ierano et al., 2014), breast cancer (Wani et al., 2014), ovarian cancer (Yu et al., 2014), glioma (Liu et al., 2013), and papillary thyroid carcinoma (Liu et al., 2014). In our current study, we found that SDF-1/CXCR7 could promote the migration and invasion ability of SGC-7901 gastric cancer cells by using a transwell chamber migration and invasion model *in vitro*.

The mechanism of tumor invasion and metastasis is very complex, and EMT has been a research hotspot in recent years. More than 90% of human malignant tumors are epithelial tumors. When EMT occurs in malignant tumor cells, it is manifested as the reduction and loss of epithelial characteristics and the acquisition of mesenchymal characteristics, ultimately enhancing the mobility and invasion ability of cells and promoting the invasion and metastasis of tumors. E-cadherin expression is downregulated in EMT, perhaps the most important change in the process, while the expression of the mesenchymal markers N-cadherin and vimentin is upregulated in EMT. The Snail transcription factor is a regulatory molecule of EMT that can downregulate E-cadherin expression by directly binding to its promoter (Chou and Yang, 2015; Ribatti et al., 2020). Studies have shown that CXCR7 promoted EMT and migration and/or invasion abilities in bladder cancer (Hao et al., 2012), lung cancer (Wu et al., 2016) and breast cancer (Li et al., 2015). Li et al. (2015) found that in breast cancer the expression of the transcription factor Snail in a CXCR7 gene silencing group after SDF-1 stimulation was reduced. Hu et al. (2014) found that the SDF-1/CXCR4 axis also significantly promoted the invasion ability of tumor cells, reduced E-cadherin expression and increased the expression of N-cadherin and vimentin, while CXCR4 also promoted the expression of Wnt pathway-related genes and target genes; blocking the Wnt pathway downregulated E-cadherin, upregulated N-cadherin and Snail, and increased the invasive ability of cells, suggesting that SDF-1/CXCR4 promotes colorectal cancer progression and EMT processes through the Wnt/β-catenin pathway. Our study showed that after the administration of SDF-1, E-cadherin expression was significantly decreased and the expression of N-cadherin, vimentin, and Snail were significantly increased. In contrast, CXCR7 knockdown increased E-cadherin level and decreased N-cadherin, vimentin and Snail levels. These results suggested that SDF-1/CXCR7 promoted EMT in gastric cancer and that the occurrence of EMT was related to the activation of Snail. The promotion of EMT by SDF-1/CXCR7 may be one of the mechanisms by which SDF-1/CXCR7 promotes the migration and invasion ability of gastric cancer cells.

In humans, matrix metalloproteinases especially MMP-2 and MMP-9 are the main gelatinases. They play key roles in the hydrolysis of the extracellular matrix and basement membrane, thus promoting tumor invasion and metastasis (Shimoda et al., 2021)_._ Li et al. (2015) found that SDF-1/CXCR7 promoted breast cancer cell invasion through increasing the expression of MMP-2 and MMP-9. Yu et al. (2014) found that SDF-1/CXCR7 induced the expression of MMP-9 through the p38 MAPK signaling pathway in ovarian cancer, thereby promoting the invasive ability of cancer cells. Our current study showed that SDF-1 could increase the expressions of MMP-2 and MMP-9 in gastric cancer cells through CXCR7, which may be another mechanism by which SDF-1/CXCR7 promotes the migration and invasion ability of gastric cancer cells. VEGF acts on endothelial cells by binding to specific receptors, thereby inducing the growth and proliferation of endothelial cells (Rajabi and Mousa, 2017), inhibiting apoptosis, promoting vascular construction, improving vascular permeability and promoting endothelial cell migration (Kieran et al., 2012). Studies have shown that SDF-1 increased VEGF mRNA expression in human umbilical vein endothelial cells and promoted VEGF- induced DNA synthesis (Neuhaus et al., 2010)_._ CXCR7 enhanced VEGFA accumulation in tumor tissues and serum of nude mice with hepatocellular carcinoma xenografts. In addition, antibody arrays and enzyme-linked immunosorbent assays were used to validate the improving effect of CXCR7 on VEGFA *in vitro* (Lin et al., 2014). Zheng et al. (2010) found that CXCR7 induced the formation of a lumen by human umbilical vein endothelial cells *in vitro* and promoted hepatocellular carcinoma angiogenesis *in vivo*. In addition, SDF-1/CXCR7 induced VEGF secretion from hepatoma cells, and VEGF positively regulated CXCR7 expression. The induction of VEGF by SDF-1/CXCR7 has also been confirmed in prostate cancer and bladder cancer (Wang et al., 2008; Hao et al., 2012). Our current study showed that SDF-1 promoted VEGF expression in gastric cancer cells through CXCR7, suggesting that SDF-1/CXCR7 may promote the angiogenesis of gastric cancer.

Although it has been confirmed that CXCR7 does not activate the G protein-dependent signaling pathway, it can recruit β-arrestin for signal transduction (Levoye et al., 2009; Rajagopal et al., 2010; Decaillot et al., 2011; Coggins et al., 2014). Our previous study showed that SDF-1 activated ERK1/2 and p38 signaling pathway in gastric cancer cells. Akt, also known as protein kinase B, is a key protein in the PI3K/Akt pathway and its activation is closely related to cell apoptosis, cell proliferation, cell cycle regulation, cell invasion and metastasis, angiogenesis and the promotion of telomerase activity in human malignancies (Cao et al., 2017; Khorasani et al., 2021). Wang et al. (2008) demonstrated that SDF-1/CXCR7 promoted Akt phosphorylation in prostate cancer. CXCR7 could also increase the p-Akt level in osteosarcoma (Zhang et al., 2014), bladder cancer (Hao et al., 2012), thyroid cancer (Zhu et al., 2016), and multiple myeloma (Azab et al., 2014). Our study also showed that SDF-1/CXCR7 increased the p-Akt level in gastric cancer cells, indicating the activation effect of SDF-1/CXCR7 on Akt signal transduction. We speculate that the activation effect of SDF-1/CXCR7 on Akt pathway may be related to the biological characteristics of promoting metastasis and EMT; however, the specific mechanism needs to be further elucidated.

## Conclusion

In summary, this study showed that SDF-1/CXCR7 enhanced the migration and invasion ability of the gastric cancer cells, increased expressions of MMP-2, MMP-9 and VEGF, promoted EMT and activated the Akt pathway. On the basis of our previous studies, these results further elucidate the mechanisms of SDF-1/CXCR7 in promoting gastric cancer progression and provide experimental basis for molecular therapy. Because CXCR7 and CXCR4 are co-expressed in gastric cancer cells and can form homodimers and heterodimers. These results also offer a basis of studying the role of SDF-1/CXCR4/CXCR7 axis in gastric cancer progress. However, we only used SGC-7901 cell line to study the mechanisms of SDF-1/CXCR7 on gastric cancer cells metastasis and only focused on the migration, invasion and EMT. In future, we will further study the underlying signal transduction and expression regulation mechanisms.

## Data Availability

The original contributions presented in the study are included in the article/supplementary material, further inquiries can be directed to the corresponding author.
